# Postoperative Adjuvant Therapy in Resectable Advanced Oral Squamous Cell Carcinoma With Intermediate Risk Factors

**DOI:** 10.1002/hed.70106

**Published:** 2025-11-27

**Authors:** Koichi Koizumi, Fumitaka Obayashi, Mirai Higaki, Kota Morishita, Atsuko Hamada, Sachiko Yamasaki, Nanako Ito, Souichi Yanamoto

**Affiliations:** ^1^ Department of Oral Oncology, Graduate School of Biomedical and Health Sciences Hiroshima University Hiroshima Japan

**Keywords:** chemoradiotherapy (CRT), intermediate risk factor, postoperative adjuvant therapy, radiotherapy (RT), resectable advanced OSCC

## Abstract

**Background:**

Postoperative adjuvant therapy strategies are generally determined based on pathological risk stratification in oral cancer. However, the efficacy of postoperative adjuvant therapy in patients with intermediate‐risk factors for recurrence of oral cancer such as close surgical margins, pT3–T4 classification, pN2–N3 nodal status, perineural invasion, vascular invasion, lymphatic invasion and pattern of invasion remains unclear, and no standardized treatment guidelines or consensus have been established to date. Therefore, this study retrospectively analyzed the clinical significance of risk factors for pathological recurrence in patients with advanced oral cancer and evaluated the impact of postoperative adjuvant therapy on patient prognosis.

**Methods:**

This retrospective clinical study included 130 patients who underwent initial radical surgical resection for advanced oral squamous cell carcinoma (OSCC) at our institution between January 2010 and December 2023. The postoperative recurrence risk factors included ENE of the cervical lymph nodes, positive surgical margins, close surgical margins, pathological T classification (pT3 or pT4), pathological N classification (pN2 or pN3), metastasis to level IV or V lymph nodes, perineural invasion, vascular invasion, and lymphatic invasion. We analyzed the relationships among the presence of these risk factors, administration of postoperative adjuvant therapy (RT or CRT), occurrence of recurrence or metastasis, and patient prognosis (DFS).

**Results:**

Patients with lymphatic invasion had a significantly lower survival rate than those without lymphatic invasion (66.7% vs. 82.8%, *p* < 0.05). Although pT4, pN2–N3, perineural invasion‐positive and vascular invasion‐positive did not reach statistical significance, a trend toward reduced survival was observed in each case. The perineural invasion‐positive group had significantly higher recurrence and metastasis rates than the perineural invasion‐negative group (51.9% vs. 23.6%, *p* < 0.05). Multivariate analysis using logistic regression also confirmed the presence of perineural invasion as an independent prognostic factor (HR = 4.496, *p* = 0.019).

**Conclusions:**

This study demonstrated that perineural invasion is a significant risk factor for recurrence and that lymphatic invasion is a poor prognostic factor in oral cancer. Postoperative adjuvant therapy in patients with perineural or lymphatic invasion‐positive disease contributed to reduced recurrence rates and prolonged survival. These findings suggest that the pathological evaluation of perineural and lymphatic invasions is an important indicator in determining the appropriateness of postoperative adjuvant therapy.

## 1 Introduction

Oral cancer is characterized by its significant local invasiveness and a high postoperative recurrence rate. Postoperative adjuvant therapy (radiotherapy (RT) and/or chemoradiotherapy (CRT)) aimed at preventing recurrence after curative resection plays a critical role in improving patient prognosis [1, 2]. Surgical resection remains the cornerstone of treatment for resectable advanced oral cancers classified as stage III or IV unless contraindicated by the patient's general condition or personal preferences. Following surgery, the intraoperative and postoperative resected specimens are evaluated to assess the risk of recurrence, which in turn determines the indication for postoperative adjuvant therapy.

Local recurrence and distant metastasis are the most significant factors influencing treatment outcomes in oral cancer. Therefore, identifying predictive factors for these outcomes is essential for optimizing therapeutic strategies [3, 4, 5, 6, 7]. The 2024 edition of the NCCN guidelines classifies recurrence risk factors into high‐ and intermediate‐risk categories. High‐risk factors include extranodal extension (ENE) of the cervical lymph nodes and positive surgical margins (R1), whereas intermediate‐risk factors comprise close surgical margins, pT3–T4 classification, pN2–N3 nodal involvement, metastasis to level IV or V lymph nodes, perineural invasion, vascular invasion, and lymphatic invasion. Among these, perineural invasion is particularly notable for its close association with tumor aggressiveness and local recurrence risk and is recognized as a poor prognostic factor. Recent studies have reported that patients with perineural invasion‐positive tumors experience higher recurrence rates and reduced disease‐free survival (DFS) and overall survival (OS) [8, 9, 10, 11, 12, 13]. However, the efficacy of postoperative adjuvant therapy in patients with perineural invasion‐positive oral cancer remains unclear, and no standardized treatment guidelines or consensus have been established to date.

Therefore, this study aimed to retrospectively analyze the clinical significance of risk factors for pathological recurrence in patients with advanced oral cancer, focusing on the presence or absence of perineural invasion. Specifically, we evaluated the impact of postoperative adjuvant therapy (RT and CRT) on patient prognosis. In doing so, we hope to contribute to the optimization of postoperative treatment strategies based on recurrence risk and provide new insights into the management of patients with perineural invasion‐positive oral cancer.

## 2 Materials and Methods

This retrospective clinical study included 130 patients who underwent initial radical surgical resection for advanced oral squamous cell carcinoma (OSCC) at our institution between January 2010 and December 2023. Advanced cases were defined as those classified as stage III or IV, according to the 8th edition of the Union for International Cancer Control Staging System. The primary tumor sites of the 130 cases were as follows: mandibular gingiva, tongue, maxillary gingiva, floor of the mouth, and buccal mucosa in 52 (40%), 33 (25%), 23 (18%), 13 (10%), and 9 (7%), respectively (Table 1A). Detailed clinicopathological characteristics of the patients are presented in Table 1B.

**TABLE 1:** Patient characteristics (A), detailed clinicopathological characteristics of the patients (B).

Characteristics		No. of cases (%)	
Surgical margin			
	Positive	17	(13.1)
	Close	11	(8.4)
	Negative	102	(78.5)
Perineural invasion			
	Positive	46	(35.4)
	Negative	84	(64.6)
Vascular invasion			
	Positive	33	(25.4)
	Negative	97	(74.6)
Lymphatic invasion			
	Positive	20	(15.4)
	Negative	110	(84.6)
Preoperative treatment			
	Yes	32	(24.6)
	No	98	(75.4)
Postoperative treatment			
	Yes	58	(44.6)
	No	72	(55.4)

Characteristics		No. of cases (%)	
Age		Av.66.14	
Gender			
	Male	87	(66.9)
	Female	43	(33.1)
Site			
	Lower gingiva	52	(40.0)
	Tongue	33	(25.4)
	Upper gingiva	23	(17.7)
	Oral floor	13	(10.0)
	Buccal mucosa	9	(6.9)
T stage			
	T1	2	(1.5)
	T2	24	(18.5)
	T3	31	(23.8)
	T4	73	(56.2)
N stage			
	N0	55	(42.3)
	N1	29	(22.3)
	N2	36	(27.7)
	N3	10	(7.8)
UICC stage			
	III	35	(26.9)
	IV	95	(73.1)

The postoperative recurrence risk factors included ENE of the cervical lymph nodes, positive surgical margins, close surgical margins, pathological T classification (pT3 or pT4), pathological N classification (pN2 or pN3), metastasis to level IV or V lymph nodes, perineural invasion, vascular invasion, and lymphatic invasion. We analyzed the relationships among the presence of these risk factors, administration of postoperative adjuvant therapy (RT or CRT), occurrence of recurrence or metastasis, and patient prognosis (DFS). The decision to initiate postoperative adjuvant therapy was based on the final pathological findings and discussion with a multidisciplinary tumor board. All enrolled patients received either RT alone or CRT, mainly with cisplatin. Recurrence or metastasis and final survival status were determined by reviewing the medical records. The primary endpoint was DFS.

### 2.1 Ethics Statement

This study was conducted in accordance with the principles of the Declaration of Helsinki and was approved by the Ethics Committee of Hiroshima University (approval number: e2023‐0056).

### 2.2 Statistical Analysis

Survival curves were generated using the Kaplan–Meier method, and between‐group differences were assessed using the log‐rank test. Multivariate analysis was performed using the Cox proportional hazards model to identify factors affecting DFS. Associations between the risk factors for recurrence, the presence or absence of postoperative adjuvant therapy, and the rates of recurrence or metastasis were evaluated using logistic regression analysis. Statistical significance was set at *p* < 0.05. All statistical analyses were conducted using JMP Pro version 16 (SAS Institute).

## 3 Results

The rates of local recurrence, cervical lymph node metastasis, and distant metastasis were 16.2%, 15.4%, and 9.2%, respectively, with an overall recurrence/metastasis rate of 36.9%. When analyzed according to the risk factors for recurrence, the high‐risk group, defined by the presence of ENE of the cervical lymph nodes and/or positive surgical margins, showed a recurrence/metastasis rate of 48.6%. The intermediate‐risk group, which included patients with close surgical margins, pT3–T4, pN2–N3 metastasis to level IV or V lymph nodes, perineural invasion, vascular invasion, or lymphatic invasion, had a recurrence/metastasis rate of 34.1%. However, the low‐risk group, with none of these pathological risk factors, had a recurrence/metastasis rate of 27.3% (Table 2).

**TABLE 2:** Postoperative treatment and recurrence/metastasis by risk factors for recurrence.

	Follow‐up observation	Re‐resection	Re‐resection + CRT (or RT)	CRT (or RT)	Subtotal	Total
High risk group						
Extranodal extension ± positive margin	1/5		0/1	10/19	11/25	18/37 (48.6%)
Positive margin		2/4	1/2	4/6	7/12	
Intermediate‐risk group						
Close margin	1/4	0/2	1/2	1/3	3/11	28/82 (34.1%)
pT3 or pT4	17/50	0/2	1/1	5/15	23/66	
pN2 or pN3	7/9			4/15	11/24	
Perineural invasion	9/18	0/1	1/2	4/6	14/27	
Vascular invasion	7/12	0/1	1/1	1/5	9/19	
Lymphatic invasion	7/11			0/1	7/12	
Low‐risk group						
No risk factors	3/11					3/11 (27.3%)
						48/130 (36.9%)

**FIGURE 1:**
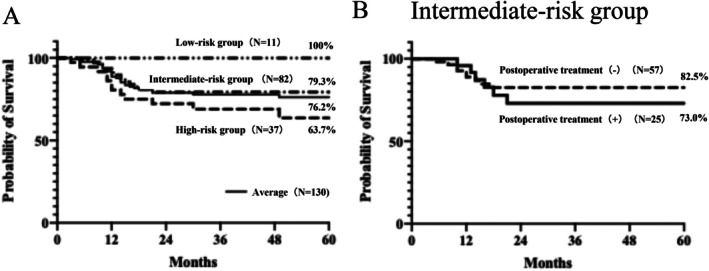
5‐year OS by risk factor for recurrence (A), 5‐year OS by intermediate risk factors for recurrence with or without postoperative treatment (B).

**FIGURE 2:**
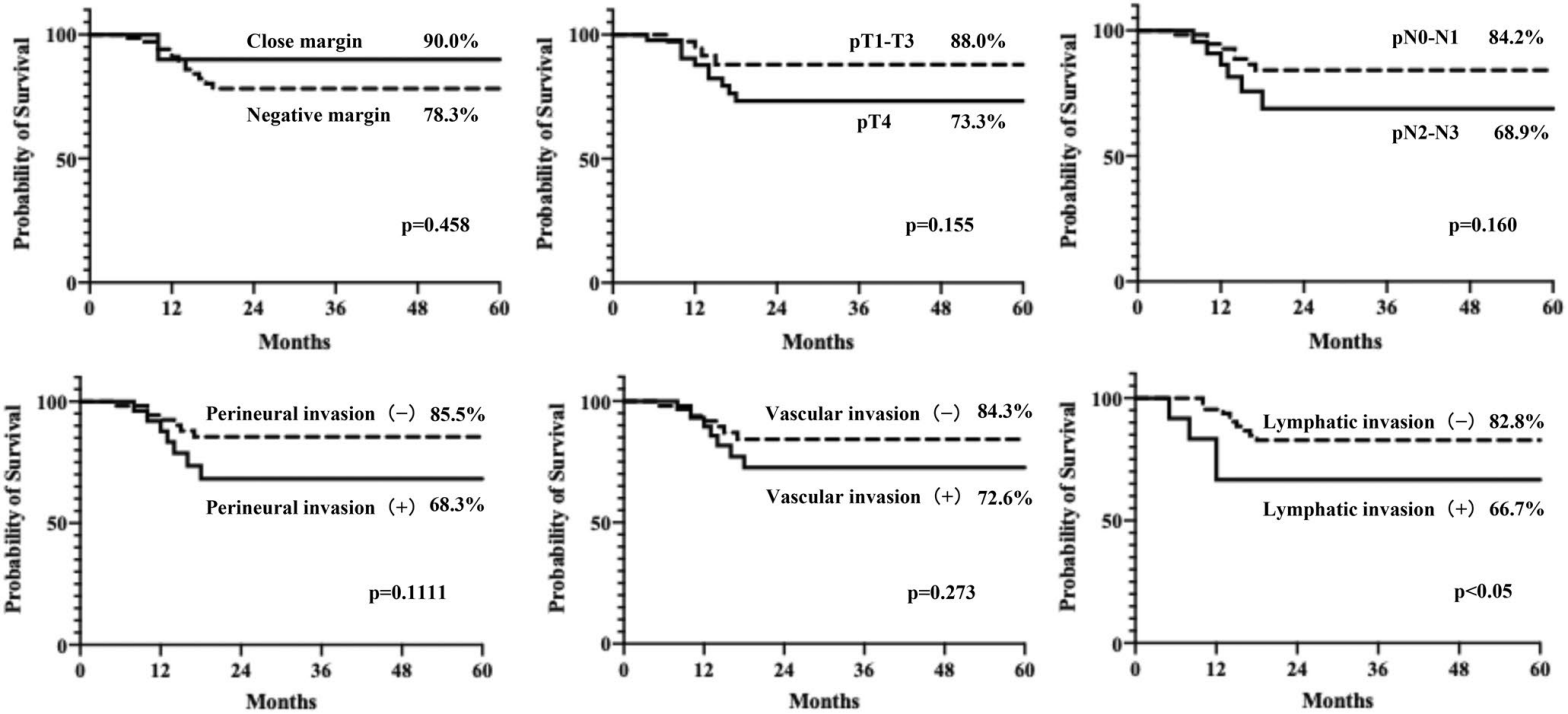
5‐year OS in the presence or absence of intermediate risk factors for recurrence.

Analysis of the association between the risk factors for recurrence and 5‐year OS showed that the high‐, intermediate‐, and low‐risk groups had a 5‐year OS rate of 63.7%, 79.3%, and 100%, respectively, with an overall average of 76.2% (Figure 1A). No significant difference in the 5‐year OS was observed between patients in the intermediate‐risk group who received postoperative therapy and those who did not (Figure 1B). When the 5‐year OS was compared according to individual intermediate risk factors, patients with lymphatic invasion had a significantly lower survival rate than those without lymphatic invasion (66.7% vs. 82.8%, *p* < 0.05). Multivariate analysis using the Cox proportional hazards model also demonstrated that the presence of lymphatic invasion was an independent prognostic factor (Hazard ratio (HR) = 3.08, *p* = 0.043), confirming its clinical relevance. Although other intermediate‐risk factors did not reach statistical significance, a trend toward reduced survival was observed in each case: pT4 versus pT1–T3 (73.3% vs. 88.0%, *p* = 0.155), pN2–N3 versus pN0–N1 (68.9% vs. 84.2%, *p* = 0.160), perineural invasion‐positive versus perineural invasion‐negative (68.3% vs. 85.5%, *p* = 0.111), and vascular invasion‐positive versus vascular invasion‐negative (72.6% vs. 84.3%, *p* = 0.273) (Figure 2, Table 3).

**TABLE 3:** 5‐year OS in the presence or absence of intermediate factors for recurrence using the Cox proportional hazards model.

	HR	95% Cl	*p*
Close margin/Negative margin	0.47	0.10–2.14	0.458
pT3‐T4/pT1‐T2	0.86	0.23–3.27	0.817
pT4/pT1‐T3	2.25	0.79–6.44	0.155
pN2‐N3/pN0‐N1	2.09	0.64–6.85	0.160
Perineural invasion (+)/(−)	2.28	0.73–7.10	0.111
Vascular invasion (+)/(−)	1.78	0.60–5.29	0.273
Lymphatic invasion (+)/(−)	3.08	0.60–15.91	< 0.05

When the recurrence and metastasis rates were compared according to individual intermediate risk factors, the perineural invasion‐positive group had a significantly higher rate than the perineural invasion‐negative group (51.9% vs. 23.6%, *p* < 0.05). Multivariate analysis using logistic regression also confirmed that the presence of perineural invasion was an independent prognostic factor (HR = 4.496, *p* = 0.019) (Table 4). Among patients with two or more intermediate risk factors, a significant difference was observed only in cases involving perineural invasion (data not shown).

**TABLE 4:** Analysis of presence/absence of intermediate risk factors for recurrence and recurrence/metastasis using logistic regression.

		Recurrence/Metastasis		HR	95% Cl	*p*
		(+)	(−)			
Close margin						
	(+)	3	8	0.719	0.128–3.246	0.678
	(−)	24	47			
pT4						
	(+)	17	27	1.763	0.695–4.655	0.234
	(−)	10	28			
pN2‐N3						
	(+)	10	14	2.937	0.812–11.296	0.101
	(−)	17	41			
Perineural invasion						
	(+)	14	13	4.496	1.276–17.786	0.019
	(−)	13	42			
Vascular invasion						
	(+)	9	10	0.665	0.148–2.643	0.571
	(−)	18	45			
Lymphatic invasion						
	(+)	7	5	3.139	0.749–14.064	0.117
	(−)	20	50			

Logistic regression analysis was used to evaluate the relationship between postoperative adjuvant therapy and recurrence/metastasis in patients with intermediate‐risk factors. The results showed a significant association only in cases with lymphatic invasion (*p* = 0.009), suggesting a potential benefit of postoperative adjuvant therapy for these patients. No significant differences in recurrence or metastasis rates were observed with or without postoperative adjuvant therapy for the other intermediate‐risk factors (Table 5).

**TABLE 5:** Analysis of postoperative treatment and recurrence/metastasis in intermediate risk factors for recurrence using logistic regression.

	Postoperative treatment	Recurrence/Metastasis				
		(+)	(−)	HR	95% Cl	*p*
Close margin (11)						
	(+)	2	5	1.200	0.076–32.783	0.898
	(−)	1	3			
pT4 (44)						
	(+)	5	5	1.833	0.431–7.882	0.431
	(−)	12	22			
pN2‐N3 (24)						
	(+)	4	11	0.181	0.026–1.021	0.053
	(−)	6	3			
Perineural invasion (27)						
	(+)	5	4	1.250	0.249–6.574	0.785
	(−)	9	9			
Vascular invasion (19)						
	(+)	2	5	0.286	0.031–1.942	0.204
	(−)	7	5			
Lymphatic invasion (12)						
	(+)	0	3			0.009
	(−)	7	2			

## 4 Discussion

Postoperative adjuvant therapy strategies are generally determined based on pathological risk stratification in oral cancer [1, 14]. The intermediate‐risk group comprises patients without high‐risk features—such as positive surgical margins or ENE of the cervical lymph nodes—who remain at risk of recurrence if treated with surgery alone. In such patients, the indication for postoperative adjuvant therapy (RT or CTR) is usually considered [15, 16, 17]. Pathological risk factors that classify a case into the intermediate‐risk category include close surgical margins, pT3–T4 classification, pN2–N3 nodal status, metastasis to level IV or V cervical lymph nodes, perineural invasion, vascular invasion, lymphatic invasion, depth of invasion (DOI) of the primary lesion, histological grade of tumor differentiation, and pattern of invasion. Determining whether adjuvant therapy should be administered to patients with these risk factors remains a significant clinical challenge, as clear and standardized criteria for such decisions have not yet been established.

Among the 37 patients with high‐risk recurrence factors, 32 (86.5%) received postoperative adjuvant therapy. Recurrence was observed in 18 (48.6%) of the patients. In contrast, within the intermediate‐risk group, defined by factors such as close surgical margins, pT3–T4 classification, pN2–N3 nodal involvement, perineural invasion, vascular invasion, and lymphatic invasion, recurrence or metastasis was observed in 28 of the 82 patients (34.1%). Notably, the recurrence rates were markedly high in patients who did not receive postoperative adjuvant therapy: 77.8% (7 of 9 cases), 50.0% (9 of 18 cases), 58.3% (7 of 12 cases), and 63.6% (7 of 11 cases) in the pN2–N3‐positive, perineural invasion‐positive, vascular invasion‐positive, and lymphatic invasion‐positive groups, respectively. These findings suggest that patients with pathological risk factors, such as pN2–N3 nodal status perineural invasion, vascular invasion, or lymphatic invasion, can benefit from a more proactive application of postoperative adjuvant therapy.

Several studies have reported differences in survival and recurrence rates based on whether postoperative adjuvant therapy was administered in patients classified as intermediate‐risk for recurrence [17, 18, 19, 20, 21]. For instance, in cases with a large DOI that resulted in disease upstaging, the OS was significantly lower in patients who did not receive postoperative RT, highlighting the importance of such therapy [22, 23, 24, 25]. Similarly, among patients with perineural invasion, those treated with surgery alone experience poorer local control and reduced DFS, whereas the addition of postoperative RT significantly improved DFS in some studies [11, 26, 27, 28]. In the present study, among patients with intermediate‐risk factors, those with lymphatic invasion showed a significantly lower 5‐year DFS than those without lymphatic invasion, suggesting the need for postoperative adjuvant therapy. Although not statistically significant, a trend toward decreased 5‐year DFS was observed in patients with pT4 tumors, pN2–N3 nodal involvement, perineural invasion positivity, or vascular invasion positivity, indicating that postoperative adjuvant therapy is also applicable in these subgroups. Conversely, the 5‐year DFS was improved in patients with close surgical margins despite the absence of postoperative adjuvant therapy, suggesting that this factor alone does not warrant such additional treatment. Multivariate analysis of factors associated with recurrence and metastasis in the intermediate‐risk group revealed that only patients with perineural invasion positivity had a significantly higher rate of recurrence and metastasis, demonstrating the presence of perineural invasion as an independent prognostic factor (HR = 4.496, *p* = 0.019). Among patients with multiple intermediate‐risk factors (i.e., having two or more of such factors), a significant difference in recurrence/metastasis was observed only in those who were perineural invasion positive (data not shown).

Analysis of the association between postoperative adjuvant therapy and recurrence/metastasis in patients with intermediate‐risk factors revealed a significant difference only in the lymphatic invasion‐positive group. These findings strongly support the recommendation to administer postoperative adjuvant therapy to patients with lymphatic invasion. RT alone or concurrent CRT is typically considered a postoperative adjuvant therapy option for patients in the intermediate‐risk groups. In patients with these pathological risk factors, postoperative RT is expected to reduce local recurrence rates and improve survival outcomes. Indeed, many retrospective studies have demonstrated a tendency for better survival outcomes in patients who received adjuvant RT compared to those who did not [17, 18, 19, 20, 21, 25]. However, the additive benefit of CRT in this risk group remains inconclusive, with different results reported across studies [2]. Therefore, the decision to administer postoperative adjuvant therapy and the specific type of treatment should be made on an individual basis, guided by the specific recurrence risk factors. Particularly, the introduction of postoperative adjuvant therapy should be strongly considered for patients with independent adverse prognostic indicators, such as lymphatic or perineural invasion. We fully acknowledge the limitation of our study's statistical power due to the relatively small sample size. In response, our findings should be interpreted with caution and that larger, multicenter, and prospective studies are warranted to validate our observations.

Each pathological risk factor classified in the intermediate‐risk group for recurrence also serves as a poor prognostic indicator, directly influencing recurrence rates and patient survival. Representative adverse prognostic factors include bone, perineural, and vascular invasion; multiple metastatic lymph nodes; close surgical margins; and poor tumor differentiation. Among them, bone invasion is a notable predictor of decreased OS [29], and perineural invasion positivity has been independently linked to poor local control and reduced DSS [26, 28]. Although the rate of distant metastasis is lower than that of local recurrence, it still has significant clinical relevance. Notably, patients with a large number of metastatic lymph nodes or extensive cervical lymph node involvement have a markedly increased risk of developing distant metastases [30, 31, 32, 33]. In the present study, 12 of the 48 patients with intermediate‐risk factors (25%) had distant metastases, and half of them had multiple lymph node metastases. These findings strongly suggest the importance and potential benefits of adjuvant therapy in patients with pN2–N3 nodal status. Therefore, evaluating recurrence and metastasis risk using a multifactorial approach, incorporating quantitative indicators, such as the extent and volume of lymph node metastasis, is essential to make informed and individualized decisions regarding the necessity and intensity of postoperative adjuvant therapy.

Based on the findings of the present study and previous reports, treatment strategies for advanced oral cancer can be summarized as follows. Surgical resection is the first‐line treatment in resectable cases. Re‐resection may be considered in some cases if postoperative pathological evaluation reveals high‐risk factors for recurrence, such as positive surgical margins or ENE of the cervical lymph nodes. However, as a general principle, postoperative CRT using RT in combination with high‐dose cisplatin is recommended. Selecting an appropriate postoperative adjuvant therapy based on specific risk factors is critical in cases with intermediate risk factors for recurrence. In particular, perineural invasion has been shown to be strongly correlated with local recurrence, and lymphatic invasion has been suggested to be associated with poorer prognoses. Therefore, postoperative RT (with the optional addition of cisplatin) should be administered in such cases. Postoperative RT should be actively considered for patients with pT4 tumors, pN2–N3 nodal involvement, or vascular invasion. Conversely, the recurrence rate is relatively low for patients who do not exhibit any of these risk factors, and the prognosis is generally favorable. In such cases, withholding postoperative adjuvant therapy and conducting careful clinical follow‐up is reasonable. Consequently, establishing personalized treatment strategies based on these risk factors is essential. Further prospective studies are necessary to optimize the treatment intensity and ensure that therapeutic interventions are tailored to each patient's profile.

In conclusion, this study demonstrated that perineural invasion is a significant risk factor for recurrence and that lymphatic invasion is a poor prognostic factor in oral cancer. Postoperative adjuvant therapy in patients with perineural or lymphatic invasion‐positive disease contributed to reduced recurrence rates and prolonged survival. These findings suggest that the pathological evaluation of perineural and lymphatic invasions is an important indicator in determining the appropriateness of postoperative adjuvant therapy. Ultimately, this study's findings could support the development of personalized treatment strategies and contribute to the optimization of therapeutic outcomes in oral cancer management.

## Funding

The authors have nothing to report.

## Ethics Statement

The authors have nothing to report.

## Conflicts of Interest

The authors declare no conflicts of interest.

## Data Availability

The data that support the findings of this study are available from the corresponding author upon reasonable request.
